# Verapamil and Alzheimer’s Disease: Past, Present, and Future

**DOI:** 10.3389/fphar.2020.00562

**Published:** 2020-05-05

**Authors:** Natalija Popović, Nicanor Morales-Delgado, David Vidal Mena, Antonia Alonso, María Pascual Martínez, María Caballero Bleda, Miroljub Popović

**Affiliations:** ^1^Department of Human Anatomy and Psychobiology, Faculty of Medicine, University of Murcia, Murcia, Spain; ^2^Institute of Biomedical Research of Murcia (IMIB), Virgen de la Arrixaca University Hospital, University of Murcia, Murcia, Spain; ^3^Department of Histology and Anatomy, Faculty of Medicine, University of Miguel Hernández, Sant Joan Alacant, Spain; ^4^Neurological Unit, University Hospital “Santa Lucia”, Cartagena, Spain; ^5^Pharmacy Unit, Nursing Home Residence “La Florida”, Alicante, Spain

**Keywords:** Alzheimer’s disease, emotion, learning, memory, verapamil

## Abstract

Verapamil is a phenylalkylamine class calcium channel blocker that for half a century has been used for the treatment of cardiovascular diseases. Nowadays, verapamil is also considered as a drug option for the treatment of several neurological and psychiatric disorders, such as cluster headache, bipolar disorders, epilepsy, and neurodegenerative diseases. Here, we review insights into the potential preventive and therapeutic role of verapamil on Alzheimer’s disease (AD) based on limited experimental and clinical data. Pharmacological studies have shown that verapamil has a wide therapeutic spectrum, including antihypertensive, anti-inflammatory, and antioxidative effects, regulation of the blood-brain barrier function, due to its effect on P-glycoprotein, as well as adjustment of cellular calcium homeostasis, which may result in the delay of AD onset or ameliorate the symptoms of patients. However, the majority of the AD individuals are on polypharmacotherapy, and the interactions between verapamil and other drugs need to be considered. Therefore, for an appropriate and successful AD treatment, a personalized approach is more than necessary. A well-known narrow pharmacological window of verapamil efficacy may hinder this approach. It is therefore important to note that the verapamil efficacy may be conditioned by different factors. The onset, grade, and brain distribution of AD pathological hallmarks, the time-sequential appearances of AD-related cognitive and behavioral dysfunction, the chronobiologic and gender impact on calcium homeostasis and AD pathogenesis may somehow be influencing that success. In the future, such insights will be crucial for testing the validity of verapamil treatment on animal models of AD and clinical approaches.

## Introduction

Alzheimer’s disease (AD) is one of the most widespread types of irreversible dementia among the older population. It is clinically characterized by a progressive cognitive decline (e.g. memory loss, difficulties in learning new tasks, problems with attention and orientation), behavioral changes (e.g. anxiety, depression, and sleep–wake cycle alterations), and inability to maintain activities of daily living (Lyketsos et al., 2011; Atri, 2019).

The early-onset form of AD (age < 65 years) is distinguished by a rapid progression and is linked to the dominant mutations in amyloid precursor protein (APP) and presenilin proteins 1 and 2 (PS 1 and PS2). Mutation in the apolipoprotein E (ApoE) gen, is related to the sporadic late-onset variation of AD (age > 65 years). This form, present in the majority of AD population, is considered multifactorial, since both genetic predisposition and several other factors contribute to the progression of the disease. Excepting aging as the major determinant, other risk factors are more modifiable including lifestyle habits (mental, physical, and social activities, smoking, nutrition), cardiovascular risk factors (obesity, diabetes mellitus, hypertension, etc.), and head injury (Hersi et al., 2017).

The main AD neuropathological features are the presence of extracellular amyloid deposits (senile plaques) in the brain parenchyma and cerebral blood vessels as well as intracellular neurofibrillary tangles accumulation (Love and Miners, 2016; Cline et al., 2018). Amyloid deposits are composed of abnormally folded amyloid β (Aβ) peptides with 40 or 42 amino acids, produced by sequential cleavage of the transmembrane APP, *via* β and ƴ-secretase enzymes. Intracellular neurofibrillary tangles are formed by an accumulation of hyperphosphorylated and misfolded tau protein. The neuropathological hallmarks are accompanied by extensive microglia cells and astrocyte activation, the key event in neuroinflammation (McGeer et al., 1989; Eikelenboom et al., 1994; Popović et al., 1998a; Henaka et al., 2015; Kim et al., 2019). These cells produce cytokines (interleukins (ILs), tumor necrosis factors (TNFs), transforming growth factors (TGFs), and interferons (IFN). In AD, some of them have pro-inflammatory (IL-1β, TNFα, and IFN-γ), anti-inflammatory (IL-4, IL-10, and TGF-β) or even both properties (IL-6) (McGeer and McGeer, 2010; Frost et al., 2019).

Both Aβ peptides and PS are implicated in the intracellular Ca^2+^ dyshomeostasis (Khachaturian, 1989; Bezprozvanny and Mattson, 2008; Demuro et al., 2010; Alzheimer’s Association Calcium Hypothesis, 2017; Pchitskaya et al., 2018). The Aβ peptides produce an excessive elevation in intracellular Ca^2+^ through the formation of Ca^2+^-permeable pores in the plasma membrane or increasing Ca^2+^ influx *via* activation of L-type Ca^2+^ channels and N-methyl-d-aspartate (NMDA) receptors. Mutated PSs may form passive Ca^2+^-leak channels on the endoplasmic reticulum, and together with enhanced ryanodine and inositol 1,4,5-trisphosphate receptors function, augment Ca^2+^ level in the cytoplasm and mitochondria (Wang and Zheng, 2019). The dysregulation of cellular Ca^2+^ homeostasis in aging and AD leads to mitochondrial dysfunction, increased production of reactive oxygen species, autophagy, impaired synaptic plasticity, reduced long-term potentiation (LTP), enhanced long-term depression, synaptic loss, cell death, and eventually cognitive decline (Disterhoft et al., 1994; LaFerla, 2002; Thibault et al., 2007; Murchison et al., 2009; Tönnies and Trushina, 2017; Peineau et al., 2018; Liu and Li, 2019). Therefore, targeting the disturbed calcium homeostasis is one plausible option for the prevention and therapy of AD.

Verapamil (C_27_H_38_N_2_O_4_), a generic name for iproveratril, which was discovered in the mid-1960s, belongs to the first generation of the phenylalkylamine class of calcium channel antagonists (Fleckenstein, 1977; Nayler and Dillon, 1986), and it is commercialized as a racemic mixture of levo (S) and dextro (R) enantiomers. Verapamil causes dilatation of the main coronary and systemic arteries and decreases myocardial contractility (Fleckenstein, 1977; Nayler and Dillon, 1986). Therefore, for many years it has been deployed as a treatment option for cardiovascular diseases, such as hypertension (Lewis et al., 1978; Midtbø et al., 1986), supraventricular tachyarrhythmias (Schamroth et al., 1972; Krikler & Spurrell, 1974), and angina pectoris (Livesley et al., 1973; Parodi et al., 1979). Remarkably, verapamil has been also used as a drug option for the treatment of hypertrophic and keloid scars (Ahuja and Chatterjee, 2014; Verhiel et al., 2015), obesity-associated autophagy defects and fatty liver pathologies (Park and Lee, 2014), osteoarthritis (Matta et al., 2015), cluster headache (Meyer and Hardenberg, 1983; Tfelt-Hansen and Tfelt-Hansen, 2009; Leone et al., 2017; Petersen et al., 2019), bipolar disorders (Wisner et al., 2002; Cipriani et al., 2016; Dubovsky, 2018), type 2 diabetes (Yin et al., 2017; Carvalho et al., 2018; Carnovale et al., 2019), chronic rhinosinusitis (Miyake et al., 2018), Peyronie’s disease (Russo et al., 2018), tuberculosis (Rayasam and Balganesh, 2015; Song and Wu, 2016), epilepsy (Nicita et al., 2016; Turner and Perry, 2017), and reversible cerebral vasoconstriction syndrome (Cappelen-Smith et al., 2017). However, verapamil has expressed ambiguous effects in Parkinson’s disease (García-Albea et al., 1993; Pasternak et al., 2012) and dementia (Maxwell et al., 1999; Yaser et al., 2005; Nimmrich and Eckert, 2013).

The aim of this mini review is to bring together data about the potential effects of verapamil on the prevention and therapy of AD, and highlights some concerns for future research.

## Alzheimer’s Disease And Verapamil

### Experimental Findings

#### *Ex Vivo* Studies

It has been demonstrated that verapamil ameliorated the neurotoxicity caused by Aβ and reduces the Aβ_1–40_ oligomer levels by improving their efflux from the LS-180 cells, *via* P-glycoprotein up-regulation (Abuznait et al., 2011). Verapamil also restores the microtubule-binding activity of tau and ameliorates the level of oxidative stress in the SH-SY5Y neuroblastoma cell line exposed to Aβ_1–42_ (Melone et al., 2018). Physiological studies indicated that the Aβ_25–35_-induced depression of LTP in a hippocampal slice preparation may be attenuated by verapamil (Freir et al, 2003). Furthermore, this drug protected MC65 neuroblastoma cells from the APP C-terminal fragment-induced neurotoxicity (Anekonda et al., 2011). However, Whitson and Appel (1995) demonstrated the inefficiency of verapamil in the prevention of Aβ_1–40_ neurotoxicity in a pure hippocampal neuronal culture, suggesting that glial-neuronal interaction is important for the preventive effect of verapamil. The rise of Ca^2+^ concentration was inhibited by verapamil in a human microglia cells culture treated with Aβ_25–35_ (Silei et al., 1999).

Verapamil pre-treatment protects IMR-32 cells against the scopolamine-induced cytotoxicity, attenuates oxidative stress, and prevents mitochondrial damage (Ponne et al., 2019). It also augments the expression of genes involved in the cholinergic function (mACR1), Ca^2+^-dependent memory-related genes (CREB1, CREBBP, BDNF), and synaptic plasticity (GAP43, SYP) that were downregulated by scopolamine (Ponne et al., 2019). Nevertheless, the lipopolysaccharide neurotoxicity in mesencephalic dopaminergic neurons that are dependent on microglia activation and augmented production of pro-inflammatory factors (superoxide, NO, and TNF-α) may be abolished by verapamil treatment (Liu et al., 2011). Similarly, verapamil significantly reduces IFN-γ;-induced neurotoxicity of human astrocytes to SH-SY5Y neuroblastoma cells (Hashioka et al., 2012). Moreover, both R and S-isomers of verapamil show equivalent potency to reduce the microglia-mediated neurotoxicity, even though the R-isomer has much less activity to block L-type calcium channels than the S counterpart (Liu et al., 2011).

#### *In Vivo* Studies

**Table 1:** Effect of verapamil (V) on learning and memory in three animal models of Alzheimer’s disease (AD): nucleus basalis magnocellularis (NBM) lesion, scopolamine (S) treatment, and intracerebroventricular injection (icv) of streptozotocin (STZ).

AD model/reference	Experimental animal	Treatment		Active avoidance		Elevated plus maze—retention	Passive avoidance—retention	Novel object recognition—retention	Morris water maze	
		Drug/dose (mg/kg)	Administration route and schedule	Acquisition	Retention				Acquisition	Retention
NBM-lesion/Popović et al., 1997a	Wistar rats Young adult ♂	Saline V 1 V 2.5 V 5 V 10	s.c., 30 min before test. Test performed 10 days after lesion.	↓ ns *p* < 0.05↑ *p* < 0.01↑ ns	↓ ns *p* < 0.05↑ *p* < 0.01↑ ns					
NBM-lesion/Popović et al., 1997c	Wistar rats Young adult ♂	Saline V 1 V 2.5 V 5 V 10	i.p., 24 h after lesion in duration of 8 days (every 12 h). Test performed 13 days after lesion.		↓ ns *p* < 0.05↑ *p* < 0.01↑ ns					
Scopolamine, 3 mg/kg i.p./Ponne et al., 2019	Swiss mice Young adult ♂	S 3 S 3+V 5 S 3+V 10	i.p., 7 days, last V dose 60 min before S and 90 min before test			↓ ns p < 0.05↑	↓ p < 0.05↑ p < 0.05↑			
Scopolamine: 0.9 mg/kg i.p., 7 days/Sekhar et al., 2016	Wistar rats Young adult ♀	S 0.9 V 21.6 V 21.6	p.o., 7 days, V 30 min: – before S – after S			↓ *p* < 0.05↑ *p* < 0.01↑		↓ *p* < 0.05↑ *p* < 0.05↑		
Scopolamine: i.p., 0.025 mg/kg, 0.05 mg/kg, 0.1 mg/kg, 1 mg/kg, 3 mg/kg/Yu et al., 2018	C57BL/6J mice Young adult ♂	S 0.025 S 0.05 S 0.1 S 1 S 3 S 0.025 + V 5 S 1 + V 5 S 3 + V 5	i.p., V 60 min before S					ns ns ns ↓ ↓ ns ns ns		
icv-STZ/Kumar et al., 2016	Wistar rats Young adult ♂	icv-STZ V 2.5 V 5	i.p. 21 days i.p. 21 days			↓ *p* < 0.05↑ *p* < 0.05↑			↓ *p* < 0.05↑ *p* < 0.05↑	↓ ns *p* < 0.05↑

s.c., subcutaneous; i.p., intraperitoneal; p.o., oral.

↑ cognitive improvement; ↓ cognitive impairment.

**Table 2:** Effect of verapamil (V) on anxiety-like behavior (open field and elevated plus maze tests), depressive-like behavior (learned helplessness, forced swimming and novelty suppressed tests) and aggression (foot-shock aggression) in two animal models of Alzheimer’s disease (AD): nucleus basalis magnocellularis (NBM) lesion and scopolamine (S).

AD model/reference	Experimental animal	Treatment		Open field				Elevated plus maze	Learned helplessness	Forced swimming test	Novelty suppressed feeding	Foot-shock aggression
		Drug/dose (mg/kg)	Administration route and schedule	Ambulation	Rearing	Activity in the center	Defecation					
NBM-lesion/Popović et al., 1997b	Wistar rats Young adult ♂	Saline V 1 V 2.5 V 5 V 10	s.c., 30 min before test. Test performed 10 days after lesion.	↑ ns *p* < 0.001↓ *p* < 0.01↓ ns	ns ns ns ns ns	↑ ns *p* < 0.001↓ *p* < 0.01↓ ns	↓ ns *p* < 0.05↑ ns ns					
NBM-lesion/Popović et al., 1997c	Wistar rats Young adult ♂	Saline V 1 V 2.5 V 5 V 10	i.p., 24h after lesion in duration of 8 days (every 12h). Test performed 13 days after lesion.	↑ ns *p* < 0.001↓ *p* < 0.001↓ ns	ns ns ns ns ns	↑ ns *p* < 0.01↓ *p* < 0.001↓ ns	↓ ns *p* < 0.001↑ *p* < 0.01↑ ns		↓ ns *p* < 0.05↑ *p* < 0.001↑ ns			↓ ns ns ns ns
NBM lesion/Popović et al., 1997c	Wistar rats Young adult ♂	Saline V 1 V2.5 V 5 V 10	s.c., 30 min before test. Test performed 10 days after lesion.						↓ ns *p* < 0.05↑ *p* < 0.05↑ ns			↓ ns ns ns ns
Scopolamine: 3 mg/kg i.p./Ponne et al., 2019	Swiss mice Young adult ♂	S 3 S 3+V 5 S 3+V 10	i.p.,7 days, last V dose 60 min before S and 90 min before test					↑ ns *p* < 0.05↓				
Scopolamine: 0.025 mg/kg i.p./Ghosal et al., 2018	Sprague-Dawley rats Young adult ♂	S 0.025 S 0.025+V 10	3 doses every other day i.p. i.p, V 30 min before S							↓ *p* < 0.05↑	↓ *p* < 0.05↑	
Scopolamine: i.p., 0.025 mg/kg, 0.05 mg/kg, 0.1 mg/kg, 1 mg/kg, 3 mg/kg/Yu et al., 2018	C57BL/6J mice Young adult ♂	S 0.025 S 0.05 S 0.1 S 1 S 3 S 0.025 + V 5 S 1 + V 5 S 3 + V 5	i.p., V 60 min before S	ns ns ns ns ns ns ns ns	ns ns ns ns ns ns ns ns					↓ ↓ ns ↑ ↑ *p* < 0.05↑ ns ns		

↑ increase values of measured behavioral parameters; ↓ decrease values of measured behavioral parameters.

Nucleus basalis of Meynert (in non-human animals correspond to the nucleus basalis magnocellularis-NBM) is composed of numerous cholinergic neurons that are affected in AD (Whitehouse et al., 1981; Rinne et al., 1987). Therefore, the lesion of this area was taken as an animal model of AD. Studies from our group with rats supported that verapamil, given in doses of 2.5 and 5 mg/kg, ameliorated the effect of NBM electrolytic lesions on learning and memory in active avoidance test (Popović et al., 1997a), anxiety behavior in open field test (Popović et al., 1997b), learned helplessness-induced depression (Popović et al., 1998b), and body temperature (Popović et al., 1998c). Findings that acute verapamil treatment (5 and 10 mg/kg), in NBM-lesioned rats, abolished cold restraint induced gastric petechiae, while in control rats, it diminished gastric erosion formation, suggest that the verapamil effect may be modified by stress (Popović et al., 1999). The long-term treatment with verapamil in NBM-lesioned rats prevented the impairments in active avoidance memory, open field behavior, and performance in learning helplessness tests (Popović et al., 1997c). Neither acute nor chronic verapamil treatment improved foot-shock induced aggression in NBM-lesioned rats (Popović et al., 1997c; Popović et al., 1998b, respectively). Moreover, morphological studies indicated that long-term verapamil treatment (2.5, 5, and 10 mg/kg/12 h) produced a significant neuroprotection on NADPH-diaphorase and ChAT-immunopositive cortical neurons in NBM lesioned rats (Caballero-Bleda et al., 2001; Popović et al., 2006). The above-mentioned studies indicated that verapamil expresses an inverted U-shape mode of action (**Tables 1** and **2**).

Systemic administration of scopolamine, a non-selective muscarinic receptor antagonist, has been considered as a pharmacological model of AD (Kwon et al., 2010; Pandareesh and Anand, 2013; Xiao et al., 2014). Long-term verapamil treatment, in a dose of 10 mg/kg (but not in a dose of 5 mg/kg), attenuated the increased mouse locomotion induced by scopolamine (Ponne et al., 2019). However, verapamil in both used doses, improved memory deficit in the elevated plus maze task and passive avoidance test, reversed the increased acetylcholinesterase (AChE) activity, and preserved the mouse brain from the oxidative stress induced by scopolamine (Ponne et al., 2019). Prophylactic and curative treatment by verapamil significantly abolished scopolamine-induced impairment in both the elevated plus maze test and the novel object recognition task in female rats (Sekhar et al., 2016). The above-mentioned treatment significantly enhanced oxidative stress markers that were decreased by scopolamine and reduced the enhanced AChE level. Furthermore, verapamil, given in a dose of 10 mg/kg, prevented the anti-depressive effect of scopolamine in rats tested in forced swim and novelty suppressed feeding tests. In the same animals, verapamil blocked the scopolamine-induced increase of the brain-derived neurotrophic factor (BDNF)/TrkB activation in the prefrontal cortex (Ghosal et al., 2018). Similarly, verapamil (5 mg/kg) significantly blockaded: 1) the scopolamine antidepressant-like effect, in mice tested in a forced swimming test; and 2) scopolamine upregulated the effect on BDNF and neuropeptide VGF (nonacronymic) content, in the hippocampus and prefrontal cortex (Yu et al., 2018). However, verapamil failed to abolish scopolamine-induced amnesia in the novel object recognition test (Yu et al., 2018) (**Tables 1** and **2**). Our recent study, focused on a passive avoidance task in rats, demonstrated that verapamil, at the dose that *per se* does not affect the memory consolidation, significantly reversed the memory consolidation improvement induced by scopolamine (Giménez de Bejar et al., 2017).

Intracerebroventricular injection (icv) of streptozotocin (STZ) has been described as an experimental model for sporadic AD (Kumar et al., 2016). In this rat model, chronic verapamil treatment (2.5 and 5 mg/kg) significantly improved memory in both the elevated plus maze test and the Morris water maze task (Kumar et al., 2016). Moreover, at mentioned doses, verapamil attenuated AChE activity in the hippocampus and frontal cortex. However, only the higher verapamil dose attenuated the oxidative stress damage and the level of TNF-α in the hippocampus and frontal cortex of icv-STZ-injected rats (Kumar et al., 2016) (**Table 1**). At the same dose, verapamil significantly reduced number of apoptotic pyramidal cells at the CA3 hippocampal region.

The icv administration of peptide Aβ_25–35_ in adult Wistar rats causes depression of LTP in the hippocampal CA1 area. This depression is significantly reversed by intraperitoneal administration of 1 mg/kg of verapamil, and with less efficacy at 10 mg/kg (Freir et al., 2003).

Verapamil (1 mg/kg/day), given orally (drinking water) for 3 months, inhibits Aβ formation and Ser202/Thr205 phosphorylation of tau by blocking the TXNIP/ROS/p38 MAPK pathway in the hippocampus of 5xFAD transgenic mice (Melone et al., 2018).

### Clinical Findings

Studies on AD patient brains have shown that phenylalkylamines receptor binding parameters, K_D_ (affinity) and B_max_ (density), are not affected in frontal, temporal, parietal, and occipital cortices, hippocampus, striatum, and thalamus (Sen et al., 1993), suggesting, altogether, that clinical trials with verapamil should not be impeded by the functionality of these receptors. One limited prospective study indicated that the use of verapamil neither significantly reduced the risk of developing AD nor had an effect on the risk of all-cause mortality (Yaser et al., 2005).

### Particularities of Mechanism of Action of Verapamil: Pros and Cons

Although initially considered as an L-type Ca^2+^ channel blocker (Cav1.2 and Cav1.3 alpha subunits), verapamil also binds the alpha subunits of P/Q type (Cav2.1), N-type (Cav2.2), R-type (Cav2.3), and T-type (Cav3.1 and Cav3.2) calcium channels (Ishibashi et al., 1995; Cai et al., 1997; Dobrev et al., 1999; Tarabova et al., 2007; Kuryshev et al., 2014). Both Cav1.2 and Cav1.3 alpha subunits are similarly upregulated at the surface level in rat cultured hippocampal neurons exposed to the Aβ_25–35_ (Kim and Rhim, 2011). On one hand, a study performed in 2, 4- and 11-month transgenic mice, overexpressing hAβPP751 with the London (V717I) and Swedish (K670M/N671L) mutations, indicated that the expression of Cav1.2 α1-subunits in reactive astrocytes, is related to the increased amyloid-β load in the plaques (Daschil et al., 2013). On the other hand, a blockade of the Cav3.1 T-type channel reduces non-amyloidogenic processing and produces higher levels of Aβ peptide in the brain of 3xTg-AD mice aged 14 to 16 months (Rice et al., 2014). The pathological, aggregated form of Aβ_1–40,_ reduced Ca^2+^ channel current density in cortical neurons, *via* an action on N-type calcium channels (Ramsden et al., 2002), as well as Aβ_1–42_ globulomer inhibited presynaptic P/Q calcium currents in hippocampal cells (Nimmrich et al., 2008). These findings pinpointed that verapamil may have a beneficial effect on L-type Ca^2+^ channels but not on P/Q-type, N-type and T-type Ca^2+^ channels.

However, verapamil, as a small conductance calcium-activated potassium channels (SK channel) antagonist (Tao et al., 2013), may have a dual effect: improve memory but fail to have a neuroprotective role in AD (Lam et al., 2013).

Besides its general action as inhibitor of the transmembrane influx of extracellular calcium ions, verapamil, in several brain regions (e.g. cerebral cortex, hippocampus, hypothalamus), acts as an antagonist of muscarinic (Baumgold, 1986; Popova et al., 1990), α- and β-adrenergic (Galzin and Langer, 1983; Staneva-Stoytcheva et al., 1990; Staneva-Stoytcheva et al., 1992), dopaminergic (Sitges and Guarneros, 1998), serotoninergic (Taylor and Defeudis, 1984; Adachi and Shoji, 1986; Green et al., 1990; Popova et al., 1991; Shad and Saeed, 2007), and GABAergic receptors (Staneva-Stoytcheva et al., 1991). Thus, it is likely from these findings and the impairments of cholinergic (Hampel et al., 2018), GABAergic (Li et al., 2016), and monoaminergic transmission (Šimić et al., 2017) in AD disease that verapamil could modify the neurotransmission in AD.

P-glycoprotein is a transmembrane glycoprotein localized in several barriers (e.g. blood-brain barrier (BBB), blood-cerebrospinal fluid barrier (BCSFB)). Its presence on the luminal membrane of the endothelial cells at the BBB (Beaulieu et al., 1997), restricts the entry of an exogenous compound (e.g., drugs, xenobiotics, toxins) into the brain and also regulates the cellular efflux from the brain. Most of the studies support that P-glycoprotein is important for Aβ_1–40_ and Aβ_1–42_ clearance, and that dysfunction of this process might lead to AD development (Lam et al., 2001; Vogelgesang et al., 2002; Vogelgesang et al., 2004; Cirrito et al., 2005; Kuhnke et al., 2007; Silverberg et al., 2010). Furthermore, microglia activation and, consequently, secretion of pro-inflammatory cytokines can disturb the P-glycoprotein production and function. On one hand, verapamil as P-glycoprotien inhibitor (Bendayan et al., 2002) may diminish the efflux of Aβ_1–40_ and Aβ_1–42_, while, on the other hand, it may restrain the pro-inflammatory processes and permit the entrance of some beneficial drugs, too.

During normal aging, there is a progressive decline in the BBB P-glycoprotein activity, particularly in men but not in women, that can lead to the accumulation of harmful compounds in the brain parenchyma (Bartels, 2011; van Assema et al., 2012a). Furthermore, the decreased BBB P-glycoprotein function in young women, compared with young men, may implicate an increased risk of AD in women (van Assema et al., 2012a). Nowadays, R-[^11^C]verapamil isomer is being used for the evaluation of the blood-brain barrier function in aging and AD, since its high uptake as radiotracer indicates decreased P-glycoprotein functionality in these conditions (Luurtsema et al., 2003; van Assema et al., 2012b; Chai et al., 2019).

Intriguingly, low and high systolic blood pressure trajectories from mid- to late-life have been associated with cognitive deficit and increased risk of AD (Skoog et al., 1996; Kivipelto et al., 2001; Qiu et al., 2004; McGrath et al., 2017). The daily fluctuations of blood pressure highlight the importance of synchronizing antihypertensive therapy with time of day (Hermida et al., 2005; Hermida et al., 2007). Similarly, the half-life of verapamil during the evening dosing regimen is much longer than the morning treatment (Jespersen et al., 1989). Bearing in mind that intracellular Ca^2+^ oscillates on a circadian and ultradian rhythm base (Noguchi et al., 2017; Wu et al., 2018) and that circadian rhythm is highly disturbed in AD patients (Coogan et al., 2013) a chronotherapy with verapamil in AD patents should be considered.

Finally, since the age and gender may modify the verapamil pharmacokinetic and pharmacodynamic effect on a stereoselective (S- and R-isomers) base (Schwartz, 1990; Sasaki et al., 1993; Schwartz et al., 1993; Schwartz et al., 1994; Gupta et al., 1995; Schwartz, 1996; Krecic-Shepard et al., 2000; Dadashzadeh et al., 2006), it seems reasonable to consider the Franco and Petrovic (2015) proposal, which suggests testing the efficacy of S- and R-verapamil molecules in AD models separately.

## Conclusion

Experimental data in animal models of AD indicated a potential positive effect of verapamil in ameliorating AD-like pathology. However, the lack of clinical findings limits its potential use in AD therapy. Probably, instead of a racemic mixture of both isomers, the equilibrate percent of each isomer should be considered. Moreover, the dose used, daytime therapy schedule, and route of verapamil application should be adjusted depending on individual conditions (gender, age, the severity of AD pathology, person’s chronotype, etc). Multimorbidity increases with aging and most of the AD patients are on polypharmacotherapy. Therefore, a possible interaction between verapamil and other drugs needs to be considered. Bearing in mind that presently, more than eight million people, just in the USA, are currently on verapamil treatment, it would seem that a rational approach to personalized AD therapy will not only be beneficial for patients, but it will also decrease the harmful impact of verapamil on the environment (Saari et al., 2017) and health economics.

## Author Contributions

All authors managed the literature searches, have read and approved the final manuscript.

## Funding

Funding was provided by the Spanish Ministry of Science, Innovation and Universities and European Regional Development Fund (FEDER; PGC2018-098229-B-100).

## Conflict of Interest

The authors declare that the research was conducted in the absence of any commercial or financial relationships that could be construed as a potential conflict of interest.
